# School Board Certificates

**Published:** 1895-12-21

**Authors:** 


### School Board Certificates.

In a leading article on this subject, the *Times* says "The London School Board has practically declared that it will not in future accept medical certificates as valid excuses for absences of children from school unless they come from medical officers appointed by itself." And further on "the only medical certificate in no case liable to be revised and set aside is one coming from a doctor specially authorised and paid by the Board."

It is only right that the public should understand the gravity of the position of affairs forced upon them by this action of the School Board. The old adage about a stitch in time applies with quite as much force to illness as it does to the mending of stockings, and while no doubt the School Board officials consider that the whole duty of woman lies in sending her children to school, people of common sense quite understand that, both for its own and for the public good, the best thing a mother can do when her child is out of sorts, not necessarily ill, is to keep it at home. If it were serious, definable illness alone which was in question, it might be another matter. But the beginnings of illness are not definable, and, except in case of fever, are often discoverable only by prolonged observation such as a mother alone can give, and yet it is the neglect of these beginnings of illness that is the cause of a great deal of the disease to which the children of the poor are so liable. If, on the other hand, we consider the end of an illness—the period of convalescence—the matter is no less serious. The very head and front of the offence charged by Mr. Sharp against the ordinary medical certificate is that sometimes it has been discovered that children certified to be unfit for school have been playing about in the streets. Where else were they to play during their convalescence? Those who have carriages and servants, and who, when their children have a little "cold," commonly enough keep them from school for weeks at a stretch, sending them out for daily walks and drives long before they allow them to be exposed to school influences, should consider carefully what convalescence too often means to the children of the poor. To them the freshest air attainable is that of the streets, and we would ask Is it seriously maintained that a child is to be kept confined to a stuffy London tenement until he is fit to stand the rough-and-tumble life of school?

A little while ago we were in a London police-court when the following dialogue took place:—

Magistrate's Clerk (addressing poor woman): Well, Mrs. ——, what about this? Hardly any attendances for the last three months.

Poor Woman: Please, sir, he's had inflammation of the lungs.

Clerk: Yes, but that was three months ago; what have you been doing with him since then?

Woman: Please, sir, he's not better yet. He's been in a convalescent home.

Clerk: Oh, yes; we know about that (reading from paper in his hand), but he came out from there more than six weeks ago.

Woman: But, sir, he ain't better yet. He's so weak, and he don't eat nothing; and he only plays a bit in the streets now and then when it's a bit fine.

Clerk: Well, but my good woman, what is to become of the boy if you don't send him to school? You must send him, you know. Have you got a doctor?

Woman: No, sir; the boy ain't ill, but he's so weak. If he's got to go I'll have to carry him myself.

Magistrate: Fortnight; medical certificate.

Policeman in charge: Come again in a fortnight with a medical certificate. Step down, please.

Now no doubt in a fortnight's time that medical certificate will be produced, and so long as such certificates are received and cases are tried before a broad-minded magistracy, appointed for other purposes and not School Board magistrates alone, the poor have some protection. But it is worthy of the most careful consideration by all who have the interests of the poor at heart, what will be the condition of affairs when that which is now but the thin end of the wedge is driven home, when in putting in force the compulsory clauses of the Education Acts, the School Board visitor, paid and dismissible by the Board, calls to his aid the School Board doctor, also paid and dismissible by the Board, and when such cases are tried (as some wish that they should be tried) by magistrates devoted to truant work alone, School Board magistrates in fact. We think that when that time comes, as it will come unless steps are taken to prevent it, the lot of the poor will be a harder one even than it is, for we cannot but see that, even now, it is only the medical profession and a common sense and independent magistracy that stand between the poor and a grinding tyranny.

